# Isolation of the Bacteriophage DinoHI from *Dichelobacter nodosus* and its Interactions with other Integrated Genetic Elements 

**DOI:** 10.2174/1874285800802010001

**Published:** 2008-01-14

**Authors:** Brian F Cheetham, Dane Parker, Garry A Bloomfield, Bruce E Shaw, Megan Sutherland, Jessica A Hyman, Jenifer Druitt, Ruth M Kennan, Julian I Rood, Margaret E Katz

**Affiliations:** 1 Molecular and Cellular Biology, University of New England, Armidale, NSW, 2351, Australia; 2 Australian Research Council Centre of Excellence in Structural and Functional Microbial Genomics and Victorian Bioinformatics Consortium, Department of Microbiology, Monash University, Clayton, Vic., 3800, Australia

## Abstract

The Gram-negative anaerobic pathogen *Dichelobacter nodosus* carries several genetic elements that integrate into the chromosome. These include the *intA*, *intB*, *intC* and *intD* elements, which integrate adjacent to *csrA *and *pnpA*, two putative global regulators of virulence and the virulence-related locus, *vrl*, which integrates into *ssrA*. Treatment of *D. nodosus* strains with ultraviolet light resulted in the isolation of DinoHI, a member of the *Siphoviridae* and the first bacteriophage to be identified in *D. nodosus*. Part of the DinoHI genome containing the packaging site is found in all *D. nodosus* strains tested and is located at the end of the *vrl*, suggesting a role for DinoHI in the transfer of the *vrl* by transduction. Like the *intB* element, the DinoHI genome contains a copy of *regA* which has similarity to the repressors of lambdoid bacteriophages, suggesting that the maintenance of DinoHI and the *intB* element may be co-ordinately controlled.

## INTRODUCTION

The Gram-negative anaerobic bacterium **Dichelobacter nodosus** is the principal causative agent of ovine footrot [1,2]. *D. nodosus* strains are classified as virulent, intermediate or benign depending on the severity of the disease which they cause in sheep. Comparative analysis of DNA from virulent and benign strains has led to the identification of a series of genetic elements which integrate into the *D. nodosus* chromosome. These include the *intA* [3-7], *intB* [8], *intC* [8] and *intD* elements [9], Tanjung, L. and Cheetham, B.F., unpublished, each of which contains an integrase gene. A fifth element, the virulence-related locus, *vrl* [6,10,11], lacks an integrase gene. The *intA *element and the *vrl* locus are found in almost all virulent strains [12,13] and are absent from the majority of benign strains.

We have proposed a model for the modulation of virulence by integration of the *intA*, *intB*, *intC* and *intD* elements into two different tRNA-ser**genes next to *csrA* (formerly *glpA*) and *pnpA*, which encode putative global regulators of virulence [14]. CsrA and the closely related protein RsmA are virulence repressors in **Salmonella enterica** [15], *Helicobacter pylori* [16], *Legionella pneumophila* [17] and *Erwinia carotovora *[18]. The *pnpA* product, polynucleotide phosphorylase, is a global regulator of virulence in *S. enterica* [19]. The *vrl* element integrates into *ssrA *[10], which encodes a tmRNA molecule that is involved in the removal of ribosomes stalled on mRNA molecules and in the subsequent proteolytic degradation of the resultant peptides [20]. In *S. enterica*, mutations in *ssrA *reduce the expression of genes from the virulence plasmid [21].

These integrated elements may have evolved from integrative plasmids or bacteriophages. To determine whether isolated a bacteriophage, DinoHI, from one strain. Cloning and analysis of the DinoHI genome showed that it was distinct from all previously-characterised integrated genetic elements of *D. nodosus*, and suggested that it may have a role in the transfer of the *vrl* locus by transduction.

## MATERIALS AND METHODOLOGY 

### General Methods

Methods for the growth of *D. nodosus*, preparation of genomic DNA, cloning and analysis of DNA, Southern blotting, DNA sequencing and DNA sequence analysis, together with the source of *D. nodosus* strains have been reported previously [7,8,14]. The nucleotide sequence of the *Nru*I-*Hin*dIII fragment containing the *intP* gene has been given the GenBank accession no. EU048235, and the junction fragment spanning *attL* in strain H1215 has been given the GenBank accession no. EU048236.

### Bacteriophage Induction – Mitomycin C

*D. nodosus* strains were inoculated into 10 ml of Eugonbroth (Becton-Dickinson) and grown under anaerobic conditions at 37PoPC for 48 h. Mitomycin C was added at varying concentrations from 0.2 to 2 μg/ml and the cultures incubated for a further 24 h. The bacterial cells were removed by centrifugation at 3000 x g for 15 min and the supernatant was filtered through a 0.45 μm HV filter (Millipore). Phage particles were collected by centrifugation at 32,000 x g for 2 h at 4PoPC and resuspended in 100 μl of SM [100 mM NaCl, 8 mM MgSOB4B, 50 mM Tris-HCl, pH 7.5, 0.01% (w/v) gelatin].

### Bacteriophage Induction – Ultraviolet Light

*D. nodosus* strains were inoculated into 10 ml of Eugonbroth and grown for 48 h. Cells were collected by centrifugation at 3,000 x g for 5 min, resuspended in 300 μl of 10 mM MgSO_4_ [22] and then the suspension was placed in a petri dish 35 cm from a 15 Watt ultraviolet germicidal lamp (intensity approximately 1000 lux) and irradiated for 10 to 20 s. The irradiated suspension was inoculated into 10 ml of Eugonbroth and incubated at 37^o^C for 24 h in the dark. Bacteriophage particles were collected by centrifugation as described above.

### Electron Microscopy

Phage preparations were diluted 1:3 in Hungate’s salts solution, then an 8 μl drop was allowed to settle for 10 min on 0.25% (v/v) formvar-coated copper mesh grid and the access liquid removed [23]. The samples were stained with either 2% (w/v) potassium phosphotungstic acid (pH 7.0) for 20 to 25 s or 4% (w/v) uranyl acetate (pH 4.0) for 5 to 10 s [24]. The prepared grids were air dried and examined using a Phillips 300 transmission electron microscope. Phage particles were photographed at a magnification of 100,000 x.

## RESULTS AND DISCUSSION

### Induction of a Bacteriophage from *D. nodosus* Strain H1215

Liquid cultures of *D. nodosus* strains A198, AC3577, C305, G1220, H1204 and H1215 were treated with mitomycin C or ultraviolet light, two commonly used agents for the induction of prophage excision [25]. These strains cover a range of *D. nodosus* serogroups and virulence classifications. After growth to stationary phase, bacterial cells were removed by centrifugation, the supernatant was subjected to high speed centrifugation to sediment bacteriophage particles, and any potential nucleic acid was extracted from the pellet. Agarose gel electrophoresis showed a band of greater than 23 kb in extracts from only one strain, H1215, and only after treatment with ultraviolet light. This result suggested that a bacteriophage had been induced. No such band was seen in uninduced cultures of strain H1215.

### Characterisation of Bacteriophage DinoHI by Electron Microscopy

**Fig. (1):**
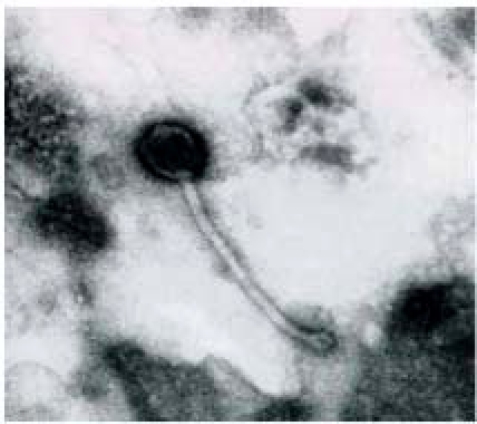
Electron micrograph of DinoHI stained with uranyl acetate. Scale bar = 50 nm.

Bacteriophage preparations for electron microscopy were negatively stained with either phosphotungstic acid or uranyl acetate and observed at a 100,000 x magnification. Phage particles (Fig. **1**) were of uniform size and comprised an icosahedral head, 57 nm apical diameter, and a tail, 10 nm x 203 nm, with a claw-like base plate, 13 nm x 15 nm. The dimensions of the tail indicate that it is non-contractile [26]. The phage head appears pentagonal, which is a distinguishing feature of icosahedral phage heads [27]. This is the first bacteriophage which has been isolated from *D. nodosus*, and it has been named DinoHI, from the host bacterial genus, species and strain [27].

### Characterisation of Phage Nucleic Acid and Classification of DinoHI

The nucleic acid isolated from the bacteriophage preparation was sensitive to DNaseI and was digested by restriction enzymes, indicating that the genome was double-stranded DNA. Pulsed field gel electrophoresis showed that the genome consisted of a single linear molecule of approximately 43 kb (data not shown). From the morphology and genome structure, DinoHI belongs to the virus family *Siphoviridae* [26].

### Restriction Enzyme Map of DinoHI

**Fig. (2):**
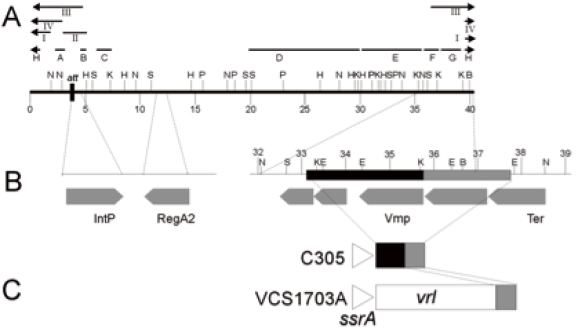
**A.** Restriction enzyme map of the linear form of the DinoHI genome. Restriction enzyme sites shown are *Bam*HI (B), *Eco*RI (E), *Hin*dIII (H), *Kpn*I (K), *Nru*I (N), *Pst*I (P) and *Sac*I (S). Distances in kb from the left hand linear end are shown on the scale line. The locations of probes A-H and fragments I-IV are shown. **B.** Expanded view of DinoHI map. Distances on the scale line are measured from the *attL* site. The integrase gene (IntP), *regA2* (RegA2), putative virion morphogeneis protein (Vmp) and large terminase subunit (Ter) genes are indicated by grey arrows. **C.** Arrangement of sequences from the DinoHI genome repeated in strains VCS1703A and C305. The diagram is not to scale.

Restriction enzyme analysis of DinoHI-derived DNA and of fragments from the DinoHI genome cloned into pUC18 was used to construct a map (Fig. **2A**). The sizes of the restriction fragments obtained by digestion of DinoHI DNA were consistent with a circular map, indicating that the linear ends of the DNA are held together tightly and are not easily separated by routine agarose gel electrophoresis (data not shown). Tight binding of fragments containing the linear ends of phage CNRZ1205 from *Streptococcus thermophilus* has been observed previously [28]. Some bands from restriction enzyme fragments spanning the unique *Bam*HI site in the DinoHI genome were present in submolar concentrations. This indicates that the linear ends are located close to this site, and there is a mixture of dissociated fragments and fragments in which the ends are still joined.

### Identification of the DinoHI Integrase Gene

The *intA, intB, intC* and *intD* elements of *D. nodosus* integrate such that the integrase gene is located approximately 200 nt from the *att* site [4,8]. A similar arrangement is seen for bacteriophages of the P4 family [29]. The *D. nodosus* integrases are related to the P4 integrase [4,8] . If the same were true for DinoHI, then the integrase gene would be found on restriction fragments which span the integration site. These fragments would be expected to be a different size in the genome of DinoHI compared to the genome of *D. nodosus* strain H1215, which contains the integrated form of the bacteriophage. Comparative Southern blot analysis of DNA from DinoHI and strain H1215 using a series of probes spanning the DinoHI genome (probes A-H in Fig. **2A**) identified several restriction fragments whose sizes differed in the genomes of DinoHI and strain H1215. Fig. (**3**) shows a sample of this data, where a 1.0 kb *Nru*I fragment hybridises to a fragment of the same size in both DinoHI and H1215 (panel A), while the adjacent 2.2 kb *Nru*I-*Hin*dIII fragment hybridises to a 2.2 kb fragment in DinoHI, but to two fragments of 1.8 kb and 1.5 kb in H1215 (panel B). Thus, the integration site was localised to this 2.2 kb *Nru*I/*Hin*dIII fragment (fragment II in Fig. **2A**), whose sequence was determined (GenBank accession no. EU048235). This fragment contained an ORF of 1197 nt, whose predicted protein product, designated IntP, has 37% amino acid identity with the integrase of a symbiosis island from the nitrogen-fixing bacterium *Mesorhizobium loti* [30]. In addition, IntP has His at position 345, Arg at 348 and Tyr at 381, which align with residues conserved between a number of bacteriophage integrases [31]. IntP has approximately 32% amino acid identity with the integrases identified in the *D. nodosus* *intA*, *intB*, *intC* and *intD* elements.

### Complete Sequence of Bacteriophage DinoHI

The sequences of both ends of twenty one subclones from the DinoHI genome were determined. While the characterisation of DinoHI was in progress, the complete sequence of the genome of *D. nodosus* strain VCS1703A was determined [32]. Blast searches of the *D. nodosus* genome sequence using the sequences from DinoHI showed that strain VCS1703A contained an integrated copy of the DinoHI genome. Sequences from DinoHI were almost identical to the corresponding sequences in the genome of strain VCS1703A, as were the restriction maps, with a small number of restriction site polymorphisms.

Analysis of the DinoHI genome revealed the presence of 47 ORFs (Table **1**). Of these genes and their gene products, 22 had no homologues in the databases, 14 had homologues of unknown function, 7 had homologues from other bacteriophages and 4 had homologues with roles in DNA replication.

### Southern Hybridisation Analysis of other Strains of *D. Nodosus*

Southern blots were carried out on genomic DNA from eighteen *D. nodosus* strains using probes A-H (Fig. **2**) spanning most of the DinoHI genome (Table **2**). Genomic DNA from six of these strains hybridised to all probes from the DinoHI genome, indicating that these strains contained a complete integrated copy of the bacteriophage. Bacteriophages were successfully induced from all of these strains after treatment with ultraviolet light. Of these strains, five are virulent and one is benign. Although almost all of the strains tested which contained DinoHI were virulent, it is unlikely that DinoHI carries genes with an essential role in virulence, as the bacteriophage is not found in strain A198, the reference virulent strain, and is also not present in the virulent strain G1220.

Genomic DNA from strain 3526 hybridised to most of the probes tested, but did not hybridise to probe B, which contains the integrase gene, *intP*, and also failed to hybridise to probe D, which spans 10 kb of the DinoHI genome. Bacteriophage particles were successfully induced from this strain, and the genome size was similar to that of DinoHI. The bacteriophage from strain 3526 appears to be related to DinoHI, but has a different integrase and other sequences which differ from DinoHI.

Genomic DNA from all strains hybridised to probes G and H, and DNA from strains C305, G1220 and H1204, which did not hybridise to most of the DinoHI probes, also hybridised to probe F. This result suggests that part of the DinoHI genome is present in all strains, and the size of this region varies between strains. Strains which contained the complete DinoHI genome had two copies of sequences detected by probes G and H, and strains which did not contain DinoHI had only one copy (data not shown). Probe G contains a phage virion morphogenesis-like protein gene, while Probe H contains part of the putative phage terminase gene (Table **1**).

### Identification of a New Integrated Genetic Element in Strain H1215

A 6 kb *Sac*I restriction fragment that contained the DinoHI integrase gene was cloned from genomic DNA of strain H1215 to construct pBSD32.4. When used to probe genomic DNA from strain H1215 digested with *Sac*I, pBSD32.4 detected a single band of 6 kb. However, when used to probe DNA from DinoHI digested with *Sac*I, it detected a band of 11 kb (fragment III, Fig. **2A**). These results indicated that pBSD32.4 spanned the site of integration, as it contained the DinoHI integrase together with chromosomal sequences from strain H1215 which are not present in the DinoHI genome.

**Fig. (4):**
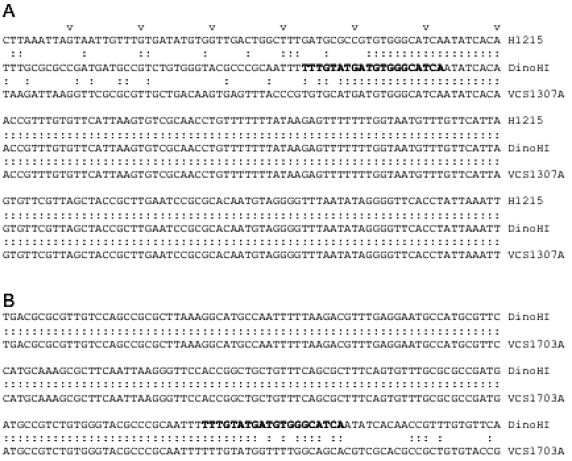
Position of *attL*. Alignment of sequences beginning 240 nt upstream from the start codon of the *intP* genes of *D. nodosus* strain H1215 (nt 340-549 of GenBank accession no. EU048236) and and VCS1703A (complement of 829317 to 829526 of GenBank accession no. CP000513) with DinoHI (nt 442 to 651 of GenBank accession no. EU048235). Every 10P^thP^ nucleotide is marked by v. Identical nucleotide sare joined by colons. **B.** Positon of *attR.* Alignment of nt 316 to 525 of DinoHI (GenBank accession no. EU048235) with the complement of nt 789133 to789342 of the VCS1703A genome (GenBank accession no. CP000513).

The sequence of part of pBSD32.4 was determined (GenBank accession no. EU048236). The DNA sequences starting 240 nt upstream from the integrase genes in pBSD32.4 (which is the integrated form in strain H1215), VCS1703A [32] and the DinoHI genome were aligned (Fig. **4A**). All three sequences differed for the first 51 nt, and were identical after this point, suggesting that the *att *site is found 189 nt upstream of the integrase gene. This is the approximate position of the *att* sites of the *intA, intB* and *intC* elements of *D. nodosus* [4,8] and of bacteriophages such as P4 [33,34]. The *att* site for DinoHI was defined as the 20 nt sequence TTTGTATGATGTGGGCATCA from DinoHI (GenBank accession no EU048235, bold in Fig. **4**) which shows 90% identity at the left junction (*attL*, upstream from *intP*) and 80% identity at the right junction (*attR*, Fig. **4B**) with genomic DNA from strain VCS1703A. Thus, the integrated DinoHI genome spans positions 829484 to 789155 in *D. nodosus *strain VCS1703A (GenBank accession no. CP000513).

**Fig. (5):**
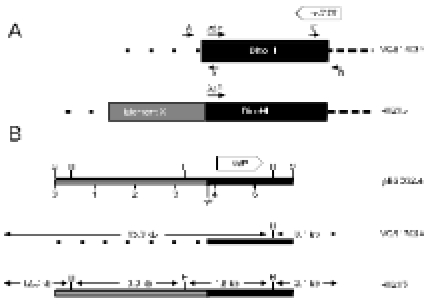
**A.** The predicted arrangement of DinoHI and Element X in the genomes of strains VCS1703A and H1215. The positions of primers A, B, C and D are shown by small arrows. The diagram is not drawn to scale. **B.** Restriction map of clone pBSD32.4 and the genomic arrangementin strains VCS1703A and H1215. Restriction sites shown are *Hin*dIII (H) and *Sac*I (S). The scale line gives distance in kb. Size sof restriction fragments in strains VCS1703A and H1215 are indicated.

The differences between the sequences on the left side of *attL* (Fig. **4A**) in strains H1215 and VCS1703A could be explained if DinoHI integrates at a different site in the genomes of these two strains. PCR primers were designed based on the genomic sequence of VCS1703A to amplify the fragments at the left and right junctions of the integrated bacteriophage (Fig. **5**). Primers C and D spanning the right hand junction amplified a fragment of the same size in strains VCS1703A and H1215, while primers A and B spanning the left hand junction amplified a fragment in VCS1703A, but not H1215 (data not shown). Sequences from pBSD32.4 on the left side of the *attL* were not detected in the VCS1703A genome by blast searches, so these sequences are absent from this strain. This may have arisen by the deletion of these sequences in strain VCS1703A. However, another possible explanation for these findings is that DinoHI is located at the same site in strains H1215 and VCS1703A, but another integrated element is present on the left hand side of DinoHI in strain H1215. The tandem integration of genetic elements has been observed previously in *D. nodosus* [8]. In addition, the sequences from pBSD32.4 which are absent from strain VCS1703A contain a type I restriction-modification system (Cheetham, B.F., Hyman, J. and Shaw, B.E., unpublished). Restriction-modification systems are often associated with mobile genetic elements [35].

The putative new integrated element in strain H1215 adjacent to DinoHI has been designated “element X” (Fig. **5A**).

The *intA, intB*, *intC* and *intD* elements of *D. nodosus* integrate into tRNA genes and the *vrl* integrates into *ssrA*. No genes encoding small RNA molecules were found to the left of the DinoHI *attL* site in VCS1703A (Fig. **4A**). However, integration of DinoHI into strain VCS1703A creates an ORF that potentially encodes a 275 aa protein (DN_0739, Table **1**) that has amino acid similarity to several secretion activator proteins [36], of which two are located on phage genomes [37,38]. The N-terminal 97 aas of this ORF are encoded to the right of *attR*, and the remaining 178 are encoded by the DinoHI genome. Homology is seen over sequences encoded both by the DinoHI genome and the chromosome.

The plasmid pBSD32.4 (Fig. **5B**), containing sequences from both DinoHI and the adjacent region (the putative element X) in strain H1215 was used to probe genomic DNA digested with *Hin*dIII from sixteen strains of *D. nodosus*. Five different patterns were seen on this Southern blot. Genomic DNA from strains A198, C305, AC3577, 819, 1169, 2483, 1493, 3138 and 1469 did not hybridise to pBSD32.4. These strains do not contain DinoHI (Table **1**), and this result suggests that they do not contain element X. There were two bands of 15 kb and 3.1 kb in DNA from strains 1311, B1006, D1172 and C1008. These strains contain DinoHI (Table **2**), but do not contain element X. DNA from strain H1215, which contains both DinoHI and element X, gave bands of 12.8, 3.1, 3.0 and 1.8 kb. Bands of 12.8, 3.0 and 1.8 kb were detected in DNA from strains G1220 and H1204, indicating that these strains contain element X, but not a complete copy of DinoHI. Finally, DNA from strain JIR1212 gave bands of 15.0, 12.8, 3.1, 3.0 and 1.8 kb. This strain appears to contain both DinoHI and element X, but the arrangement differs from strain H1215. One possible explanation is that there is a copy of DinoHI at each end of element X. Thus, element X was present in only four out of sixteen strains tested. Of these, three were virulent and one was benign.

### A Copy of *regA* in the DinoHI Genome

The *intB *element of *D. nodosus* contains a gene, *regA*, which is related to several regulatory proteins, including the cI repressor from lambdoid bacteriophages [8]. Blast search analysis of the DinoHI genome showed that it also contained a copy of *regA*, designated *regA2* (Fig. **2A**). Comparison of the sequences from the *intB* element of *D. nodosus* strain A198 and the DinoHI genome revealed a 701 nt sequence with 97% identity. This identity begins immediately after the start codon of the *intB *element *regA*, and ends 25 nt after the stop codon. The predicted aa sequence for the two products is 100% identical for 231 of the 232 amino acids of the *intB* *regA*. However, *regA2* lacks the start codon found in *regA*. Instead, there are two potential start codons upstream in the same reading frame, which would result in an additional 17 or 144 aa at the N-terminus. The predicted length of the *intB*-encoded RegA protein is very close to that of closely-related proteins identified through blast searches, typically 230-231 aa.

The high level of nt identity between *regA* and *regA2* suggests a recent duplication, or a selective mechanism for the maintenance of the DNA sequence. Analysis of the junction regions did not reveal any special features such as repeated sequences. By contrast, the *intA* element contains repeats of a 102 nt and a 103 nt repeat and some duplicated sequences from the *intA* element are bounded by these repeats [9].

Expression of *regA* and *regA2* would lead to the production of two proteins which are almost identical, except for an additional sequence at the N-terminus in RegA2. These proteins are highly related at the aa level to repressor proteins of lambdoid bacteriophages, which act to maintain these phages in the integrated state. It is possible that RegA maintains the *intB *element in an integrated state, and RegA2 maintains the DinoHI prophage. However, the high structural similarity could allow co-ordinate control of these two genetic elements, as each repressor may act on both integrated elements.

### Mapping of the Linear Ends of DinoHI

Southern blots of DinoHI DNA digested with several restriction enzymes and probed with DNA from several regions of the DinoHI genome showed the presence of faint bands (data not shown), consistent with pieces containing the linear ends. In particular, DinoHI DNA digested with KpnI and EcoRV and probed with a 2.1 kb BamHI/NruI fragment (probe I, Fig. **2**) detected a strong band of 4.3 kb (fragment IV, Fig. **2**) and two faints bands of 3.0 and 1.3 kb, localising the linear end to approximately 0.4 kb from the BamHI site, about 37,000 nt from the att site (Fig. **2A**). This site is immediately downstream from the terminase gene, and in several other lambdoid bacteriophages the linear ends are located close to the terminase gene [28,39,40]. At position 36952 in DinoHI, the sequence TCGGGGCGGCG is found, which perfectly matches part of the bacteriophage lambda cos site [41]. An 11 nt sequence is repeated at positions 36748 and 36897, resembling the arrangement of repeat sequences which direct the nicking of lambda DNA at the cos site [42]. Thus, the Southern blot evidence and sequence information support the location of the linear ends of DinoHI at this position.T

### The DinoHI Packaging Site is Found at the End of the *vrl* Region

Southern blot analysis (Table **1**) showed that all strains hybridised to probes G and H (Fig. **2A**), even if they did not contain the rest of the DinoHI genome, and some strains lacking DinoHI also hybridised to probe F. This was investigated by Blast searches using the DinoHI genome sequence, numbered from the beginning of the attachment site immediately upstream from *intP*. Nt 1-40318 of the DinoHI genome correspond to nt 829484-789167 of the *D. nodosus* strain VCS1703A genome. Nt 35837-37637 of the DinoHI genome were found to be present at the end of the *vrl* in strain VCS1703A and A198 [10]. The linear ends of DinoHI have been mapped to within 100 bp of position 37,000 (Fig. **2B**), which is within this repeated fragment. T The presence of the DinoHI packaging site at the end of the *vrl* suggests that the *vrl* region may be transferred between T*D. nodosus* strains by transduction. Alternatively, the *vrl* may be a prophage that has an identical packaging site to that of DinoHI.

Genomic DNA from strain C305, which lacks the *vrl* region, hybridises to probes F, G and H. A 3.5 kb fragment was amplified from strain C305 [10], using primers from within *ssrA* and within the region of DinoHI which is repeated at the end of the *vrl* (Fig. **2C**). Sequencing of the ends of this fragment indicated that the fragment contains nt 33053-35837 from DinoHI. Thus, there is a 4.6 kb segment of DinoHI, which includes the packaging site, next to *ssrA* in strain C305. This arrangement could have been generated by integration of DinoHI into *ssrA*, followed by deletion of most of the DinoHI genome. However, *ssrA* is not the integration site for DinoHI in either strain H1215 or VCS1703A. This segment of DinoHI contains a gene involved in virion morphogenesis and part of the terminase gene. The significance of this arrangement is not clear.

## CONCLUSIONS 

**Fig. (6):**
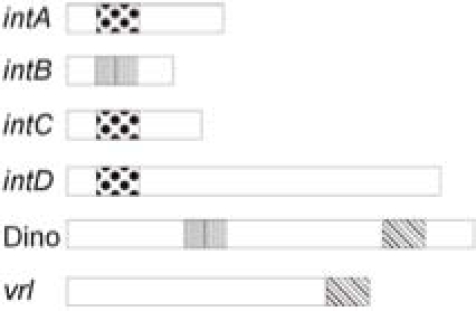
Common regulatory sequences in the integrated elements of *D. nodosus.* The positions of *vap*H/*vap*G (dotted), *regA* (verticalbars) and the DinoHI packaging site (45P^oP^ bars) in the *intA*, *intB*,*intC* and *intD* elements, the *vrl* and DinoHI are shown. The diagram is not drawn to scale.

We have identified a new bacteriophage, DinoHI, with an integrase that is very similar to the integrases in the *intA*, *intB*, *intC* and *intD* elements. The discovery that the packaging site of DinoHI is located at the end of the *vrl *region (Fig. **6**) suggests that the *vrl* may be transferred by transduction.

The finding that the DinoHI genome contains a copy of *regA*, a gene found on the *intB* element and encoding a protein with similarity to the repressors of lambdoid phages, suggests that there may be coordinate regulation of the maintenance of the *intB* element and DinoHI. Similarly, the *intA*, *intC* and *intD* elements all have genes designated **vap*G* and **vap*H*, (Fig. **6**) whose arrangement resembles that of the immunity region of bacteriophage P4 [43]. In P4, this immunity region contributes to the maintenance of the bacteriophage in an integrated state, and prevents superinfection. Thus, the maintenance of the *intA*, *intC* and *intD* elements may be also co-ordinately regulated. Altogether, these results suggest that the integrated elements of *D. nodosus* are not independent, but instead are part of a complex regulatory system which controls the expression of virulence genes.

## Figures and Tables

**Fig. (3):**
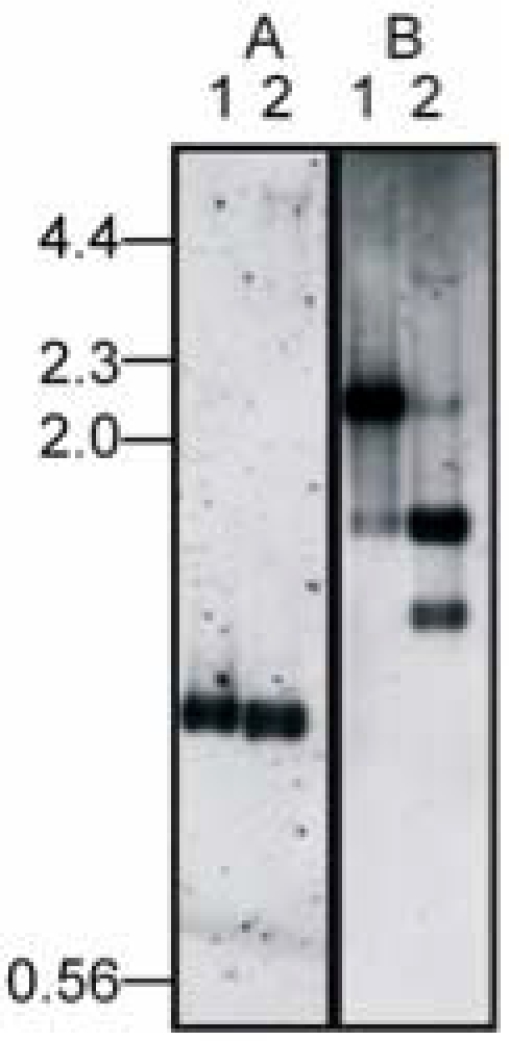
Identification of integration site. Genomic DNA from DinoHI (lane 1) and *D. nodosus* strain H1215 (lane 2) was digested with *Nru*I (panel A) or *Nru*I and *Hin*dIII (panel B) and probed in panel A with a 1.0 kb *Nru*I fragment from DinoHI (probe A, Fig.**1A**) or in panel B with a 2.2 kb *NruI-Hind*III from DinoHI (fragmentII, Fig. **2A**). Sizes in kb of molecular weight markers are shown on the left hand side.

**Table 1:** Putative ORFs from DinoHI. (c)hp = (Conserved)Hypothetical Protein. Coordinates are Taken from GenBank Accession no. CP000513

Start	Stop	Product/gene
788865	789692	chp - membrane
789685	789960	hp
789863	790168	hp
790165	790956	hp
790934	792208	phage terminase, large subunit
792205	793662	chp, pseudo
793659	795158	phage virion morphogenesis-like protein
795669	796067	hp
796078	796899	hp
796902	797888	chp
797910	798377	chp
798374	798799	phage virion morphogenesis-like protein
798780	799247	hp
799244	800011	chp
800014	800241	hp
800249	800458	hp
800495	804466	phage tail tape measure family protein
804463	804867	chp
804876	808505	chp
808507	809373	chp
809285	809710	hp
809707	811383	chp
811392	811619	hp
812618	812055	hp
814279	812666	helicase family protein
815422	814328	hp
816242	815574	hp
816617	816363	hp
817090	816635	single-strand binding protein/*ssb*
817960	817103	chp
818562	817957	bacteriophage recombination function protein
818920	818588	hp
819207	818920	hp
820748	819315	chp
821794	820877	regulatory protein/s*regA2*
822196	822459	hp
822460	823347	chp
823352	824116	DNA replication protein/*dnaC*
824871	825815	chp
824363	825878	hp
825812	826231	hp
826234	826611	endodeoxyribonuclease RusA family
826605	826961	hp
826961	827212	chp
827196	827603	hp

**Table 2:** Hybridisation of Genomic DNA from *D. Nodosus* Strains to Probes from the DinoHI Genome (Fig. 2A). + Indicates Hybidisation, - Indicates no Hybridisation

Strain	Virulence	Probe							
		A	B	C	D	E	F	G	H
A198	Virulent	-	-	-	-	-	-	+	+
B1006	Virulent	+	+	+	+	+	+	+	+
C1008	Virulent	+	+	+	+	+	+	+	+
D1172	Virulent	+	+	+	+	+	+	+	+
G1220	Virulent	-	-	-	-	-	+	+	+
H1215	Virulent	+	+	+	+	+	+	+	+
1311	Virulent	+	+	+	+	+	+	+	+
3526	Virulent	+	-	+	-	+	+	+	+
AC3577	Intermediate	-	-	-	-	-	-	+	+
AC390	Benign	+	+	+	+	+	+	+	+
C305	Benign	-	-	-	-	-	+	+	+
H1204	Benign	-	-	-	-	-	+	+	+
819	Benign	-	-	-	-	-	-	+	+
1169	Benign	-	-	-	-	-	-	+	+
1469	Benign	-	-	-	-	-	-	+	+
1493	Benign	-	-	-	-	-	-	+	+
2483	Benign	-	-	-	-	-	-	+	+
3138	Benign	-	-	-	-	-	-	+	+
